# Association of Sociodemographic and Health-Related Factors With Receipt of Nondefinitive Therapy Among Younger Men With High-Risk Prostate Cancer

**DOI:** 10.1001/jamanetworkopen.2020.1255

**Published:** 2020-03-19

**Authors:** Alexander F. Bagley, Mitchell S. Anscher, Seungtaek Choi, Steven J. Frank, Karen E. Hoffman, Deborah A. Kuban, Sean E. McGuire, Quynh-Nhu Nguyen, Brian Chapin, Ana Aparicio, Todd A. Pezzi, Grace L. Smith, Benjamin D. Smith, Kenneth Hess, Chad Tang

**Affiliations:** 1 Department of Radiation Oncology, The University of Texas MD Anderson Cancer Center, Houston; 2 Department of Urology, The University of Texas MD Anderson Cancer Center, Houston; 3 Department of Genitourinary Medical Oncology, The University of Texas MD Anderson Cancer Center, Houston; 4 Department of Health Services Research, The University of Texas MD Anderson Cancer Center, Houston; 5 Department of Statistics, The University of Texas MD Anderson Cancer Center, Houston

## Abstract

**Importance:**

Multiple randomized clinical trials have shown that definitive therapy improves overall survival among patients with high-risk prostate cancer. However, many patients do not receive definitive therapy because of sociodemographic and health-related factors.

**Objective:**

To identify factors associated with receipt of nondefinitive therapy (NDT) among patients aged 70 years and younger with high-risk prostate cancer.

**Design, Setting, and Participants:**

This cohort study identified 72 036 patients aged 70 years and younger with high-risk prostate cancer and Charlson Comorbidity Index scores of 2 or less who were entered in the National Cancer Database between January 2004 and December 2014. Data analysis was conducted from November 2018 to December 2019.

**Exposure:**

Receipt of NDT as an initial treatment approach.

**Main Outcomes and Measures:**

Survival rates were compared based on receipt of definitive therapy or NDT, and sociodemographic and health-related factors were associated with the type of therapy received. Residual life expectancy was estimated from the National Center for Health Statistics to calculate person-years of life lost.

**Results:**

A total of 72 036 men with a median (range) age of 63 (30-70) years, Charlson Comorbidity Index scores of 2 or less, and high-risk prostate cancer without regional lymph node or distant metastatic disease were analyzed. Among eligible patients, 5252 (7.3%) received NDT as an initial therapeutic strategy. On univariate and multivariate analyses, NDT was associated with worse overall survival (univariate analysis hazard ratio, 2.54; 95% CI, 2.40-2.69; *P* < .001; multivariate analysis hazard ratio, 2.40; 95% CI, 2.26-2.56; *P* < .001). Compared with patients with private insurance or managed care, those with no insurance, Medicaid, or Medicare were more likely to receive systemic therapy only (no insurance: odds ratio [OR], 3.34; 95% CI, 2.81-3.98; *P* < .001; Medicaid: OR, 2.92; 95% CI, 2.48-3.43; *P* < .001; Medicare: OR, 1.36; 95% CI, 1.20-1.53; *P* < .001) or no treatment (no insurance: OR, 2.63; 95% CI, 2.24-3.08; *P* < .001; Medicaid: OR, 1.71; 95% CI, 1.45-2.01; *P* < .001; Medicare: OR, 1.14; 95% CI, 1.04-1.24; *P* = .004). Compared with white patients, black patients were more likely to receive systemic therapy only (OR, 1.93; 95% CI, 1.74-2.14; *P* < .001) or no treatment (OR, 1.46; 95% CI, 1.32-1.61; *P* < .001), and Hispanic patients were more likely to receive systemic therapy only (OR, 1.36; 95% CI, 1.13-1.64; *P* = .001) or no treatment (OR, 1.36; 95% CI, 1.14-1.60; *P* < .001). Between 2004 and 2014, patients without insurance or enrolled in Medicaid had 1.83-fold greater person-years of life lost compared with patients with private insurance (area under the curve, 77 600 vs 42 300 person-years of life lost).

**Conclusions and Relevance:**

In this study, receipt of NDT was associated with insurance status and race/ethnicity. While treatment decisions should be individualized for every patient, younger men with high-risk prostate cancer and minimal comorbidities should be encouraged to receive definitive local therapy regardless of other factors. These data suggest that significant barriers to life-extending treatment options for patients with prostate cancer remain.

## Key Points

**Question:**

What factors are associated with the receipt of nondefinitive therapy and survival among patients aged 70 years and younger who are diagnosed with high-risk prostate cancer?

**Findings:**

In this cohort study of 72 036 patients aged 70 years and younger with high-risk prostate cancer entered in the National Cancer Database, we found that 7.3% of patients received nondefinitive therapy; moreover, receipt of nondefinitive therapy was associated with inferior overall survival. Insurance status and race/ethnicity were independently associated with receipt of nondefinitive therapy.

**Meaning:**

The findings of this study suggest that significant barriers to life-extending treatment options for younger patients with high-risk prostate cancer remain.

## Teaser

This cohort study identifies sociodemographic and health-related factors associated with receipt of nondefinitive therapy among patients aged 70 years and younger with high-risk prostate cancer.

## Introduction

High-risk prostate cancer represents approximately 15% of newly diagnosed prostate cancer cases in the United States annually, corresponding to more than 26 000 patients.^1,2^ The current standard of care options for definitive therapy include radical prostatectomy (RP), external beam radiotherapy (EBRT), or brachytherapy-based radiotherapy with or without EBRT, often in combination with androgen-deprivation therapy (ADT). Nondefinitive therapy (NDT) may include systemic therapy (ie, ADT or chemotherapy) or no initial therapy (ie, active surveillance or watchful waiting). Evidence from several randomized clinical trials and retrospective studies demonstrates that, compared with ADT, definitive local therapy improves overall survival in men with high-risk prostate cancer.^3,4,5,6,7,8,9^ Based on these data, the current National Comprehensive Cancer Network and American Urological Association/American Society for Radiation Oncology/Society of Urologic Oncology guidelines recommend NDT only for high-risk or very high-risk patients with limited life expectancy (ie, ≤5 years).^10,11^

However, a substantial number of men aged 70 years and younger do not receive definitive local therapy despite a estimated life expectancy of more than 14 years, based on Social Security actuarial tables.^12,13,14,15,16^ Moreover, men aged 70 years who are in the lowest quartile of overall health because of medical comorbidities have an adjusted life expectancy of 8 years and would therefore still meet the current guidelines recommending definitive local therapy.^17^ Using the National Cancer Database (NCDB), we analyzed the patterns of care for younger (ie, ≤70 years) men with high-risk prostate cancer to identify factors associated with receipt of NDT and to determine the association of NDT with survival. We hypothesized that a significant number of patients with high-risk prostate cancer who are younger and have fewer medical comorbidities continue to receive NDT and, moreover, that receipt of NDT in this population is associated with inferior survival.

## Methods

This analysis was reviewed by the institutional review board of the University of Texas MD Anderson Cancer Center, which gave prior approval and waived written informed consent for this retrospective analysis using fully deidentified data. This report followed the Strengthening the Reporting of Observational Studies in Epidemiology (STROBE) reporting guideline for cohort studies.

We identified patients entered in the NCDB from January 2004 to December 2014 diagnosed with high-risk prostate cancer, as defined by the National Comprehensive Cancer Network risk groupings (ie, T stage 3 or 4, prostate-specific antigen [PSA] >20 ng/mL [to convert to micrograms per liter, multiply by 1.0], or Gleason score of 8-10). The NCDB is a joint project of the American Cancer Society and the Commission on Cancer of the American College of Surgeons. The American College of Surgeons has a data-use agreement with each of its Commission on Cancer–accredited hospitals. The NCDB, established in 1989, is a nationwide, facility-based, comprehensive clinical surveillance resource oncology data set that currently captures 70% of all newly diagnosed malignant neoplasms in the United States annually.

We abstracted the following sociodemographic and disease-related variables from our data set to define risk groups and perform regression analyses: insurance status, income level, race/ethnicity, age, education level, great circle distance between the patient’s residence and the hospital that reported the case, facility type, urban/rural group, clinical TNM stage, tumor histology, PSA level, Gleason score, and Charlson Comorbidity Index score. To determine treatment groups, we abstracted the following variables: type of radiation for primary and boost treatments, total dose of radiation, type of surgery, type of chemotherapy, type of hormone therapy, and receipt of palliative care.

Inclusion criteria were as follows: aged 70 years and younger, Charlson Comorbidity Index score of 2 or less, clinical N0 (or NX) and M0 disease, and adenocarcinoma histology. Life expectancy in the absence of cancer was estimated using Social Security actuarial tables and models adjusting for coexisting medical comorbidities.^8,9,10,11,12,13^ Definitive therapy was defined as receipt of RP, EBRT (≥60 Gy), or brachytherapy-based treatment (including monotherapy or boosts with EBRT ≥45 Gy), with or without ADT. Nondefinitive therapy was defined as systemic therapy only (ie, hormone therapy or chemotherapy) or no treatment (ie, active surveillance or no administered therapy). Exclusion criteria were receipt of nononcologic local tumor excision or receipt of ablative procedures, including cryoablation.

### Statistical Analysis

We calculated person-years of life lost (PYLLs) at each age (single years from age 30 to 70 years) as the product of the number of deaths within a given age and subgroup and the residual life expectancy in the absence of cancer, as described previously.^18,19^ Absolute PYLLs were summed over all age groups to calculate total PYLLs. To compare PYLLs among different treatments and insurance statuses, we had to accommodate considerable differences in the absolute number of patients within each subgroup. Therefore, we calculated an adjusted, or normalized, PYLL by dividing the PYLL at each age by the total number of patients within that subgroup and age. Adjusted PYLLs were averaged for each age group (ie, ≤50 years, 51-55 years, 56-60 years, 61-65 years, 66-70 years, and ≥71 years) per 1000 persons and plotted using Prism version 8.0.0 (GraphPad) software.

To reduce immortal time bias, we excluded patients in the NDT groups with follow-up duration of less than the 90th percentile of the time for patients to begin definitive treatment after diagnosis (ie, 3.9 months). Multivariable analysis was performed using logistic regression and Cox proportional hazards models. Stepwise selection with a significance level of α < .05 was used for selection and removal of variables from the regression models. Survival analysis was performed using the Kaplan-Meier method, with log-rank tests used to compare survival among groups. Statistical analysis was performed using SAS statistical software version 9.4 (SAS Institute). Statistical significance was set at α < .05, and all tests were 2-tailed.

## Results

### Patient, Treatment, and Disease Characteristics

**Figure 1.**
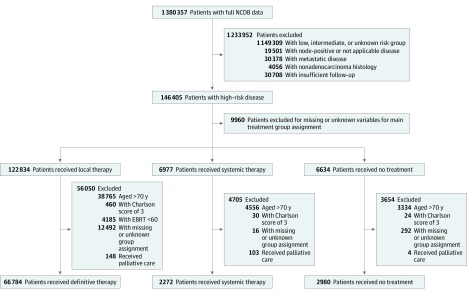
Flow Diagram of Patient Eligibility and Inclusion EBRT indicates external beam radiation therapy; NCDB, National Cancer Database.

We analyzed 72 036 men with a median (range) age of 63 (30-70) years with high-risk prostate cancer who met inclusion eligibility criteria (Figure 1). A total of 26 404 patients (36.7%) were aged 60 years or younger, 22 010 (30.6%) were aged 61 to 65 years, and 23 622 (32.8%) were aged 66 to 70 years. Among all patients, 70 580 (98.0%) had Charlson Comorbidity Index scores of 0 to 1. Based on actuarial estimates and adjusting for comorbidities, the anticipated life expectancies for these patients were at least 13.9 years.^17^ Overall, 5252 patients (7.3%) received NDT as their initial treatment. Certain sociodemographic subgroups received NDT at higher rates than the overall NDT rate, including uninsured patients (450 of 2078 [21.7%]), Medicaid patients (485 of 2689 [18.0%]), black patients (1738 of 13 591 [12.8%]), Hispanic patients (354 of 3114 [11.4%]), and patients treated in a community cancer program (683 of 5916 [11.5%]). Patients receiving NDT were categorized as systemic therapy only (2260 of 2272 [99.5%] receiving hormone therapy; 12 [0.5%] receiving chemotherapy) or no treatment (473 of 2980 [15.9%] receiving active surveillance; 2507 [84.1%] receiving no therapy). Among patients receiving systemic therapy only, 846 (37.2%) had stage T3 or T4 disease, while among those receiving no treatment, 242 (8.1%) had stage T3 or T4 disease. Additional baseline patient and treatment characteristics are shown in Table 1.

**Table 1.** Patient, Treatment, and Disease Characteristics

Characteristic	Patients, No. (%)^a^			
	Overall (N = 72 036)	Definitive therapy (n = 66 784)	Nondefinitive Therapy (n = 5252)	
			Systemic only (n = 2272)	No treatment (n = 2980)
Age, y				
≤60	26 404 (100)	24 606 (93.2)	705 (2.7)	1093 (4.1)
61-65	22 010 (100)	20 393 (92.7)	690 (3.1)	927 (4.2)
66-70	23 622 (100)	21 785 (92.2)	877 (3.7)	960 (4.1)
Race/ethnicity				
White, non-Hispanic	49 829 (100)	46 437 (93.2)	1744 (3.5)	1648 (3.3)
Black	13 601 (100)	11 863 (87.2)	861 (6.3)	877 (6.4)
Hispanic/Spanish	3113 (100)	2759 (88.6)	159 (5.1)	195 (6.3)
Asian	1316 (100)	1200 (91.2)	51 (3.9)	65 (4.9)
Other or unknown	4212 (100)	3863 (91.7)	154 (3.7)	195 (4.6)
Insurance status				
Not insured	2077 (100)	1627 (78.3)	210 (10.1)	240 (11.6)
Private insurance or managed care	38 422 (100)	35 809 (93.2)	1257 (3.3)	1356 (3.5)
Medicaid	2694 (100)	2209 (82.0)	267 (9.9)	218 (8.1)
Medicare	25 844 (100)	23 667 (91.6)	1129 (4.4)	1048 (4.1)
Other government	2027 (100)	1952 (96.3)	42 (2.1)	33 (1.6)
Unknown	1007 (100)	858 (85.2)	64 (6.4)	85 (8.4)
Income level, $^b^				
<38 000	13 280 (100)	11 789 (88.8)	759 (5.7)	732 (5.5)
38 000-47 999	16 794 (100)	15 319 (91.2)	758 (4.5)	717 (4.3)
48 000-62 999	18 757 (100)	17 367 (92.6)	695 (3.7)	695 (3.7)
≥63 000	22 751 (100)	21 225 (93.3)	722 (3.2)	804 (3.5)
Not available	489 (100)	422 (86.3)	35 (7.2)	32 (6.5)
Population without high school degree, %				
>21	12 198 (100)	10 799 (88.5)	711 (5.8)	688 (5.6)
13-20.9	18 549 (100)	16 910 (91.2)	795 (4.3)	844 (4.6)
7.0-12.9	22 984 (100)	21 374 (93.0)	849 (3.7)	761 (3.3)
<7.0	17 885 (100)	16 654 (93.1)	584 (3.3)	658 (3.8)
Not available	444 (100)	385 (86.7)	30 (6.8)	29 (6.5)
Facility type				
Community cancer program	5921 (100)	5238 (88.5)	312 (5.3)	371 (6.3)
Comprehensive community cancer program	30 809 (100)	29 012 (94.2)	919 (3.0)	878 (2.8)
Academic or research program	27 959 (100)	25 042 (89.6)	1460 (5.2)	1457 (5.2)
Integrated network cancer program	7351 (100)	6804 (92.6)	276 (3.8)	271 (3.7)
Other or unknown	31 (100)	26 (83.9)	2 (6.5)	3 (9.7)
Urban/rural group^c^				
Metropolitan	57 667 (100)	52 872 (91.7)	2358 (4.1)	2437 (4.2)
Urban	10 997 (100)	10 136 (92.2)	458 (4.2)	403 (3.7)
Rural	1581 (100)	1456 (92.1)	72 (4.6)	53 (3.4)
Other or unknown	1826 (100)	1658 (90.8)	81 (4.4)	87 (4.8)
Great circle distance, miles				
≤4.9	17 366 (100)	15 631 (90.0)	836 (4.8)	899 (5.2)
5.0-11.4	17 662 (100)	16 213 (91.8)	671 (3.8)	778 (4.4)
11.5-27.9	18 317 (100)	16 947 (92.5)	690 (3.8)	680 (3.7)
≥28.0	18 181 (100)	16 852 (92.7)	741 (4.1)	588 (3.2)
Other or unknown	545 (100)	479 (87.9)	31 (5.7)	35 (6.4)
Definitive therapy				
Radical prostatectomy	39 695 (55.1)	39 695 (59.4)	NA	NA
EBRT	19 496 (27.1)	19 496 (29.2)	NA	NA
Brachytherapy based	7593 (10.5)	7593 (11.3)	NA	NA
Systemic therapy				
Hormone therapy	2260 (3.1)	NA	2260 (99.5)	NA
Chemotherapy	12 (<0.1)	NA	12 (0.5)	NA
No treatment				
Active surveillance	473 (0.7)	NA	NA	473 (15.9)
No therapy administered	2507 (3.5)	NA	NA	2507 (84.1)
Charlson Comorbidity Index score				
0	60 434 (100)	55 977 (92.6)	1845 (3.1)	2612 (4.3)
1	10 146 (100)	9484 (93.5)	343 (3.4)	319 (3.1)
2	1456 (100)	1323 (90.9)	84 (5.8)	49 (3.4)
Clinical T stage				
1	36 807 (100)	33 494 (91.0)	1196 (3.2)	2117 (5.8)
2	20 324 (100)	18 779 (92.4)	924 (4.5)	621 (3.1)
3	13 928 (100)	13 070 (93.8)	672 (4.8)	186 (1.3)
4	866 (100)	636 (73.4)	174 (20.1)	56 (6.5)
Unknown	146 (100)	143 (97.9)	3 (2.1)	0
Gleason Score				
6	4416 (100)	3734 (84.6)	83 (1.9)	599 (13.6)
7	8883 (100)	8238 (92.7)	308 (3.5)	337 (3.8)
8	18 888 (100)	17 952 (95.0)	540 (2.9)	396 (2.1)
9	11 415 (100)	10 646 (93.3)	545 (4.8)	224 (2.0)
10	789 (100)	681 (86.3)	81 (10.3)	27 (3.4)
Other or unknown	27 680 (100)	24 871 (89.9)	1412 (5.1)	1397 (5.0)

Abbreviations: EBRT, external beam radiotherapy; NA, not applicable.

^a^ Data represent number of patients and corresponding row percentages (%) except for treatment characteristics in which column percentages are shown.

^b^ Median household income for patient’s area of residence.

^c^ Data estimated by population of patient’s county using data from 2013 published by United States Department of Agriculture Economic Research Service. Metropolitan represents counties in metropolitan areas with a population range of fewer than 250 000 to greater than 1 million; urban represents populations of 2500 to greater than 20 000, either adjacent or not adjacent to a metropolitan area; rural represents completely rural populations or areas with less than 2500 residents.

### Association of NDT With Overall Survival

**Figure 2.**
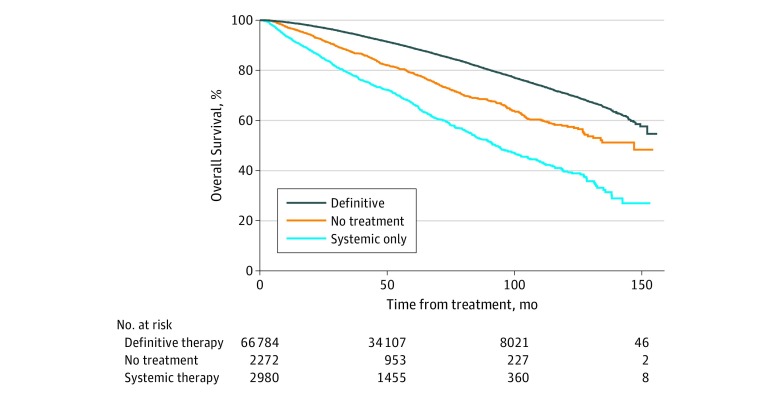
Survival of Patients Aged 70 Years and Younger With High-Risk Prostate Cancer, Based on Receipt of Definitive Therapy, Systemic Therapy Only, or No Treatment

At a median follow-up of 54.9 months (95% CI, 54.6-55.2 months), the overall survival rates were 91.3% (95% CI, 91.1%-91.6%), 83.5% (95% CI, 81.9%-85.0%), and 69.8% (95% CI, 67.5%-71.9%) for definitive therapy, no therapy, and systemic therapy only, respectively (*P* < .001) (Figure 2). On univariate analyses, NDT was associated with worse overall survival (hazard ratio [HR], 2.54; 95% CI, 2.40-2.69; *P* < .001). On multivariate analysis (factoring in variables including insurance status, age group, race/ethnicity, income and educational levels, treatment facility type, distance from treatment facility, and disease characteristics), NDT was significantly associated with worse overall survival (HR, 2.40; 95% CI, 2.26-2.56; *P* < .001) (Table 2). Within the NDT subgroup, systemic therapy only (HR, 3.06; 95% CI, 2.83-3.31; *P* < .001) and no active initial treatment (HR, 1.85; 95% CI, 1.69-2.02; *P* < .001) were each independently associated with worse overall survival.

**Table 2.** Factors Associated With Overall Survival for Patients With High-Risk Prostate Cancer^a^

Characteristic	Univariate Analysis		Multivariate analysis	
	HR (95% CI)	*P* value	HR (95% CI)	*P* value
Treatment group				
Definitive therapy	1 [Reference]	NA	1 [Reference]	NA
Nondefinitive therapy	2.54 (2.40-2.69)	<.001	2.40 (2.26-2.56)	<.001
Systemic therapy	3.70 (3.44-3.98)	<.001	3.06 (2.83-3.31)	<.001
No treatment	1.77 (1.63-1.93)	<.001	1.85 (1.69-2.02)	<.001
Insurance Status				
Private insurance or managed care	1 [Reference]	NA	1 [Reference]	NA
Not insured	1.80 (1.61-2.02)	<.001	1.36 (1.20-1.54)	<.001
Medicaid	2.38 (2.16-2.61)	<.001	1.88 (1.69-2.08)	<.001
Medicare	1.84 (1.76-1.92)	<.001	1.42 (1.34-1.50)	<.001
Other government	1.53 (1.34-1.74)	<.001	1.50 (1.30-1.72)	<.001
Income level, $^b^				
≥63 000	1 [Reference]	NA	1 [Reference]	NA
48 000-62 999	1.31 (1.23-1.39)	<.001	1.13 (1.05-1.21)	.001
38 000-47 999	1.53 (1.44-1.62)	<.001	1.21 (1.12-1.31)	<.001
<38 000	1.77 (1.67-1.88)	<.001	1.23 (1.12-1.35)	<.001
Race/ethnicity				
White, non-Hispanic	1 [Reference]	NA	1 [Reference]	NA
Black	1.31 (1.25-1.38)	<.001	1.07 (1.01-1.14)	.02
Hispanic/Spanish	0.85 (0.76-0.96)	.01	0.65 (0.57-0.73)	<.001
Asian	0.70 (0.58-0.84)	<.001	0.71 (0.58-0.87)	.001
Age, y				
≤60	1 [Reference]	NA	1 [Reference]	NA
61-65	1.39 (1.32-1.47)	<.001	1.27 (1.19-1.34)	<.001
66-70	1.78 (1.69-1.87)	<.001	1.40 (1.31-1.50)	<.001
Population without high school degree, %				
<7.0	1 [Reference]	NA	1 [Reference]	NA
7.0-12.9	1.28 (1.21-1.36)	<.001	1.11 (1.04-1.20)	.003
13-20.9	1.50 (1.41-1.59)	<.001	1.18 (1.09-1.28	<.001
≥21	1.73 (1.62-1.84)	<.001	1.23 (1.12-1.35)	<.001
Clinical T stage				
1	1 [Reference]	NA	1 [Reference]	NA
2	1.27 (1.21-1.33)	<.001	1.29 (1.22-1.36)	<.001
3	1.25 (1.19-1.32)	<.001	1.39 (1.31-1.47)	<.001
4	3.75 (3.37-4.17)	<.001	3.09 (2.73-3.49)	<.001
Charlson Comorbidity Index score				
0	1 [Reference]	NA	1 [Reference]	NA
1	1.39 (1.32-1.47)	<.001	1.28 (1.20-1.36)	<.001
2	2.42 (2.17-2.70)	<.001	2.11 (1.87-2.37)	<.001
Great circle distance, miles				
≤4.9	1 [Reference]	NA	1 [Reference]	NA
5.0-11.4	0.88 (0.83-0.93)	<.001	0.97 (0.91-1.03)	.26
11.5-27.9	0.83 (0.79-0.88)	<.001	0.88 (0.83-0.94)	<.001
≥28.0	0.78 (0.73-0.82)	<.001	0.78 (0.72-0.84)	<.001
Facility type				
Academic or research program	1 [Reference]	NA	1 [Reference]	NA
Community cancer program	1.50 (1.40-1.61)	<.001	1.18 (1.09-1.29)	<.001
Comprehensive community cancer program	1.20 (1.15-1.26)	<.001	1.16 (1.10-1.22)	<.001
Integrated network cancer program	1.14 (1.06-1.23)	<.001	1.11 (1.03-1.21)	.008
Urban/rural group^c^				
Metropolitan	1 [Reference]	NA	1 [Reference]	NA
Urban	1.17 (1.11-1.24)	<.001	1.10 (1.02-1.18)	.01
Rural	1.29 (1.14-1.47)	<.001	1.21 (1.05-1.40)	.01

Abbreviations: HR, hazard ratio; NA, not applicable.

^a^ A Cox regression model was used for univariate and multivariate analyses.

^b^ Median household income for patient’s area of residence.

^c^ Data estimated by population of patient’s county using data from 2013 published by US Department of Agriculture Economic Research Service. Metropolitan represents counties in metropolitan areas with a population range of fewer than 250 000 to greater than 1 million; urban represents populations of 2500 to greater than 20 000, either adjacent or not adjacent to a metropolitan area; and rural represents completely rural populations or areas with less than 2500 residents.

### Association of Insurance Status With Receipt of NDT and PYLLs

On multivariate analysis including sociodemographic, health-related, and disease-related factors, the most significant sociodemographic factor associated with receipt of NDT was the patient’s insurance status (Table 3). Compared with those with private insurance or managed care, patients with no insurance, Medicaid, or Medicare were more likely to receive systemic therapy only (no insurance: odds ratio [OR], 3.34; 95% CI, 2.81-3.98; *P* < .001; Medicaid: OR, 2.92; 95% CI, 2.48-3.43; *P* < .001; Medicare: OR, 1.36; 95% CI, 1.20-1.53; *P* < .001) or no treatment (no insurance: OR, 2.63; 95% CI, 2.24-3.08; *P* < .001; Medicaid: OR, 1.71; 95% CI, 1.45-2.01; *P* < .001; Medicare: OR, 1.14; 95% CI, 1.04-1.24; *P* = .004).

**Table 3.** Factors Associated With Nondefinitive Therapy for Patients With High-Risk Prostate Cancer^a^

Characteristic	Nondefinitive therapy compared with systemic therapy				Nondefinitive therapy compared with no treatment			
	Univariate		Multivariate		Univariate		Multivariate	
	OR (95% CI)	*P* value	OR (95% CI)	*P* value	OR (95% CI)	*P* value	OR (95% CI)	*P* value
Insurance status								
Private insurance or managed care	1 [Reference]	NA	1 [Reference]	NA	1 [Reference]	NA	1 [Reference]	NA
Not insured	4.66 (3.96-5.48)	<.001	3.34 (2.81-3.98)	<.001	3.46 (3.00-4.00)	<.001	2.63 (2.24-3.08)	<.001
Medicaid	4.43 (3.81-5.14)	<.001	2.92 (2.48-3.43)	<.001	2.37 (2.04-2.74)	<.001	1.71 (1.45-2.01)	<.001
Medicare	1.73 (1.57-1.90)	<.001	1.36 (1.20-1.53)	<.001	1.14 (1.05-1.24)	.002	1.14 (1.04-1.24)	.004
Other government	0.63 (0.43-0.93)	.02	0.52 (0.35-0.78)	.002	0.46 (0.32-0.65)	<.001	0.38 (0.26-0.56)	<.001
Income level, $^b^								
≥63 000	1 [Reference]	NA	1 [Reference]	NA	1 [Reference]	NA	NI	NI
48 000-62 999	1.32 (1.16-1.49)	<.001	1.25 (1.09-1.43)	.002	1.05 (0.94-1.16)	.41	NI	NI
38 000-47 999	1.78 (1.58-2.02)	<.001	1.59 (1.40-1.82)	<.001	1.20 (1.09-1.33)	<.001	NI	NI
<38 000	2.44 (2.16-2.75)	<.001	1.70 (1.49-1.95)	<.001	1.57 (1.42-1.74)	<.001	NI	NI
Race/ethnicity								
White, non-Hispanic	1 [Reference]	NA	1 [Reference]	NA	1 [Reference]	NA	1 [Reference]	NA
Black	2.42 (2.21-2.65)	<.001	1.93 (1.74-2.14)	<.001	2.00 (1.84-2.17)	<.001	1.46 (1.32-1.61)	<.001
Hispanic/Spanish	1.91 (1.60-2.28)	<.001	1.36 (1.13-1.64)	.001	1.95 (1.67-2.27)	<.001	1.36 (1.14-1.60)	<.001
Asian	1.14 (0.81-1.59)	.46	1.06 (0.75-1.48)	.76	1.52 (1.18-1.96)	.001	1.19 (0.91-1.54)	.20
Age, y								
≤60	1 [Reference]	NA	1 [Reference]	NA	1 [Reference]	NA	NI	NI
61-65	1.17 (1.05-1.30)	.004	1.19 (1.06-1.34)	.003	1.01 (0.93-1.11)	.80	NI	NI
66-70	1.39 (1.25-1.53)	<.001	1.44 (1.27-1.64)	<.001	0.97 (0.89-1.06)	.46	NI	NI
Population without high school degree, %								
<7.0	1 [Reference]	NA	NI	NI	1 [Reference]	NA	1 [Reference]	NA
7.0-12.9	1.30 (1.14-1.47)	<.001	NI	NI	0.89 (0.80-0.99)	.04	0.89 (0.80-1.00)	.05
13-20.9	1.66 (1.46-1.89)	<.001	NI	NI	1.24 (1.12-1.37)	<.001	1.13 (1.00-1.26)	.05
≥21	2.44 (2.14-2.78)	<.001	NI	NI	1.54 (1.38-1.72)	<.001	1.18 (1.04-1.34)	.01
Clinical T stage								
1	1 [Reference]	NA	1 [Reference]	NA	1 [Reference]	NA	1 [Reference]	NA
2	1.30 (1.18-1.44)	<.001	1.36 (1.22-1.51)	<.001	0.52 (0.47-0.57)	<.001	0.53 (0.48-0.58)	<.001
3	1.23 (1.10-1.37)	<.001	1.39 (1.24-1.57)	<.001	0.22 (0.19-0.26)	<.001	0.23 (0.20-0.27)	<.001
4	6.29 (5.20-7.62)	<.001	6.23 (5.06-7.66)	<.001	1.04 (0.80-1.37)	.76	1.02 (0.76-1.36)	.90
Charlson Comorbidity Index score								
0	1 [Reference]	NA	1 [Reference]	NA	1 [Reference]	NA	1 [Reference]	NA
1	1.10 (0.98-1.23)	.12	1.02 (0.90-1.15)	.81	0.71 (0.63-0.80)	<.001	0.70 (0.62-0.79)	<.001
2	1.90 (1.52-2.38)	<.001	1.59 (1.24-2.02)	<.001	0.76 (0.57-1.01)	.06	0.70 (0.52-0.95)	.02
Great circle distance, miles								
≤4.9	1 [Reference]	NA	NI	NI	1 [Reference]	NA	1 [Reference]	NA
5.0-11.4	0.73 (0.65-0.82)	<.001	NI	NI	0.85 (0.77-0.93)	.001	0.93 (0.84-1.03)	.18
11.5-27.9	0.64 (0.57-0.72)	<.001	NI	NI	0.71 (0.64-0.79)	<.001	0.84 (0.75-0.94)	.002
≥28.0	0.72 (0.64-0.81)	.001	NI	NI	0.62 (0.56-0.69)	<.001	0.70 (0.62-0.79)	<.001
Facility type								
Academic or research program	1 [Reference]	NA	1 [Reference]	NA	1 [Reference]	NA	1 [Reference]	NA
Community cancer program	1.29 (1.13-1.48)	<.001	1.14 (0.99-1.31)	.07	1.19 (1.06-1.34)	.004	1.13 (1.00-1.28)	.06
Comprehensive community cancer program	0.65 (0.59-0.72)	<.001	0.67 (0.60-0.74)	<.001	0.53 (0.49-0.58)	<.001	0.54 (0.49-0.59)	<.001
Integrated network cancer program	0.82 (0.70-0.95)	.007	0.79 (0.68-0.93)	.003	0.69 (0.60-0.79)	<.001	0.70 (0.60-0.80)	<.001
Urban/rural group^c^								
Metropolitan	1 [Reference]	NA	NI	NI	1 [Reference]	NA	NI	NI
Urban	1.06 (0.94-1.18)	.36	NI	NI	0.86 (0.77-0.96)	.006	NI	NI
Rural	1.27 (0.99-1.65)	.07	NI	NI	0.79 (0.60-1.04)	.09	NI	NI

Abbreviations: NA, not applicable; NI, not included; OR, odds ratio.

^a^ A logistic regression model was used for univariate and multivariate analyses.

^b^ Median household income for patient’s area of residence.

^c^ Data estimated by population of patient’s county using data from 2013 published by US Department of Agriculture Economic Research Service. Metropolitan represents counties in metropolitan areas with a population range of fewer than 250 000 to greater than 1 million; urban represents populations of 2500 to greater than 20 000, either adjacent or not adjacent to a metropolitan area; and rural represents completely rural populations or areas with less than 2500 residents.

Additional sociodemographic factors associated with receipt of NDT included race/ethnicity, the median household income level in the patient’s county of residence, and treatment facility type. Compared with white patients, black patients were more likely to receive systemic therapy only (OR, 1.93; 95% CI, 1.74-2.14; *P* < .001) or no treatment (OR, 1.46; 95% CI, 1.32-1.61; *P* < .001), and Hispanic patients were more likely to receive systemic therapy only (OR, 1.36; 95% CI, 1.13-1.64; *P* = .001) or no treatment (OR, 1.36; 95% CI, 1.14-1.60; *P* < .001). Patients living in counties with lower median household incomes (ie, <$38 000 per year) were more likely to receive systemic therapy only compared with those living in counties with a median income of at least $63 000 per year (OR, 1.70; 95% CI, 1.49-1.95; *P* < .001). On univariate analysis, patients treated at community cancer programs were more likely to receive systemic therapy alone than those treated at academic or research programs (OR, 1.29; 95% CI, 1.13-1.48; *P* < .001). Uninsured black and Hispanic patients received NDT at rates of 23.9% (185 of 772) and 23.7% (66 of 278) respectively, while uninsured white patients received NDT at a rate of 16.9% (150 of 887). We found considerable heterogeneity in treatment patterns across individual treatment facilities associated with treatment facility type and the number of patients treated per facility (eFigure 1 and eFigure 2 in the Supplement). Additional associations of these factors with receipt of NDT are included in Table 3.

Between 2004 and 2014, patients without insurance or receiving Medicaid had 1.83-fold greater PYLL compared with patients with private insurance (area under the curve, 77 600 vs 42 300 PYLL). Within each group younger than 70 years, PYLL was greater among patients without insurance or receiving Medicare compared with patients with private insurance, with the largest difference observed in the group aged 51 to 55 years (area under the curve, 3900 vs 1820 PYLL; 2.14-fold greater PYLL for patients without insurance or receiving Medicaid vs private insurance) (eFigure 3 in the Supplement).

### Health-Related and Disease-Related Factors Associated With Receipt of NDT

Although patients had life expectancies warranting definitive therapy based on current national guidelines, we investigated whether specific health-related and disease-related factors were associated with the decision to offer NDT. Tumor stage and Charlson Comorbidity Index score were independently associated with increased rates of NDT. On multivariate analysis, patients with tumor stages T2, T3, and T4 were more likely to receive systemic therapy only compared with patients with stage T1 disease (T2: OR, 1.36; 95% CI, 1.22-1.51; *P* < .001; T3: OR, 1.39; 95% CI, 1.24-1.57; *P* < .001; T4: OR, 6.23; 95% CI, 5.06-7.66; *P* < .001). Furthermore, patients with a Charlson Comorbidity Index score of 2 were more likely to receive systemic therapy only than patients with a Charlson Comorbidity Index score of 0 (OR 1.59; 95% CI, 1.24-2.02; *P* < .001). Additional factors associated with receipt of NDT and definitive therapy are included in Table 3 and the eTable in the Supplement.

## Discussion

This study examined the patterns of care and survival outcomes among patients aged 70 years and younger with high-risk prostate cancer and Charlson Comorbidity Index scores of 2 or less who were entered in the NCDB from 2004 to 2014. This population was selected because patients had an estimated life expectancy greater than 5 years and therefore warranted definitive therapy based on the currently accepted national guidelines. Our primary conclusions are as follows: (1) NDT was associated with worse overall survival; (2) the most significant factors associated with receiving NDT were insurance status, race/ethnicity, median household income, and health-related and disease-related factors, including tumor stage and medical comorbidity score; (3) the association between receiving NDT and sociodemographic factors was comparable to associations with health-related and disease-related factors; and (4) the relative PYLLs were greater among patients without insurance or with Medicaid than among those with private insurance. While it may be justified to withhold definitive local therapy for selected patients with significant medical comorbidities, most patients in this analysis (ie, 98%) had a medical comorbidity score of 0 or 1 and life expectancies of 13.9 years or greater. While policies, including the Patient Protection and Affordable Care Act, expanded health care access in the United States, this finding suggests that significant barriers to life-extending treatment options for younger patients with high-risk prostate cancer remain.

A second finding is that patients receiving systemic therapy had worse survival compared with patients receiving no treatment. This may be explained in part by patients receiving systemic therapy having more bulky disease that is not captured in the available staging. The current National Comprehensive Cancer Network guidelines for patients with high-risk, nonmetastatic cancer recommend systemic therapy for symptomatic patients.^11^ Among patients receiving systemic therapy only, 37.2% (ie, 846 of 2272) had stage T3 to T4 disease, whereas for those receiving no treatment, 8.1% (242 of 2980) had stage T3 to T4 disease. While we excluded patients with metastatic disease in this analysis, we found that bulky primary disease (ie, T3 or T4) was independently associated with worse survival, regardless of treatment type.

Insurance status was significantly associated with receipt of NDT, which was in turn associated with decreased survival. This association was demonstrated in our comparison of PYLLs among those without insurance or receiving Medicaid vs those with private insurance. Person-years of life lost is a useful metric in quantifying the consequences of cancer-related deaths, particularly in studies of younger populations who would otherwise have longer life expectancies.^18,19^ We found that patients without insurance had greater PYLLs for all age groups examined (ie, aged ≤70 years), with the largest difference occurring in the group aged 51 to 55 years. Multiple factors in addition to insurance status may be associated with PYLLs, including receipt of NDT, Charlson Comorbidity Index score, and clinical T stage, which on multivariate analysis were each independently associated with decreased overall survival.

Data from randomized clinical trials have established an overall survival benefit for high-risk patients treated with definitive radiotherapy and ADT compared with ADT alone. The SPCG-7/SFUO-3 trial^3^ randomized patients to lifelong ADT vs combination definitive pelvic radiotherapy and lifelong ADT, and with a median follow-up of 7.6 years, the 10-year incidence of death was 23.9% and 11.9%, respectively. The MRC PR07 trial^5^ similarly randomized patients to lifelong ADT vs combination pelvic radiotherapy and lifelong ADT and reported improved 7-year disease-specific survival (81% vs 90%) and overall survival (66% vs 74%) with inclusion of definitive local therapy. Consistent with these randomized studies, we conclude that NDT is associated with worse overall survival, independent of health-related and sociodemographic factors.

Regarding the association of treatment patterns with treatment facility, we observed considerable heterogeneity in the rates of NDT across individual facilities, and treatment facility type was independently associated with receipt of NDT. Our analysis revealed certain high-performing facilities that offered definitive therapy at higher rates than was seen for the overall population. While the identities of individual facilities cannot be determined from NCDB analyses, future efforts to evaluate the practices of high-performing facilities may identify quality improvement strategies to reduce rates of NDT, particularly among patient subgroups at a higher risk of receiving NDT. A 2019 study demonstrated that an electronic medical record–based intervention was successful in narrowing racial disparities associated with completing treatment of early-stage cancers.^20^ Similar approaches may be warranted to reduce the overall rates and sociodemographic disparities associated with receiving NDT for high-risk prostate cancer.

Our results expand on previous studies reporting patterns of care for patients with prostate cancer. Wong et al^14^ analyzed patients younger than 60 years with low-, intermediate-, and high-risk localized prostate cancer entered in the Surveillance, Epidemiology, and End Results database and reported that 15.9% of high-risk patients received no active treatment; furthermore, black patients, uninsured patients, or patients receiving Medicaid were less likely to receive definitive treatment.^14^ Burt et al^15^ analyzed all risk groups and observed that, while patients younger than 65 years with localized prostate cancer were more likely to undergo RP than older patients, RP was less commonly used among black patients, uninsured patients, or patients receiving Medicaid.^15^ Mahal et al^13^ studied the associations of advanced age and black race with the receipt of definitive therapy for patients with high-risk prostate cancer entered in the Surveillance, Epidemiology, and End Results database and observed that black patients were less likely to receive definitive therapy than white patients, a disparity more prominent among older patients.^13^ To our knowledge, the current study is the first to exclusively focus on younger patients with high-risk disease, and by using the NCDB, we were able to separate definitive radiation doses from palliative doses and obtain information on patients’ medical comorbidities. Collectively, these studies and our data highlight ongoing disparities in care associated with sociodemographic factors such as race/ethnicity and insurance status.

### Limitations

There are several potential limitations to the current study. First, the validity of our conclusions is based on the accuracy of data entered in the NCDB, including nodal status and Gleason score; while prior studies have examined the accuracy of large cancer registries, such as the Surveillance, Epidemiology, and End Results database, for prostate cancer–specific variables and treatments, we are not aware of specific studies that directly confirm the accuracy of these variables in NCDB in the setting of high-risk prostate cancer.^21,22^ Second, we excluded patients who received less than 60 Gy of EBRT without a documented radiation boost, but some of these patients may have received additional therapeutic interventions, such as a brachytherapy boost, that were not recorded in the initial treatment course. Third, patients included in the NDT groups may have received definitive therapies later in their treatment course or at medical centers different from where they were initially diagnosed, but this information is not available given that only the first treatment course is recorded. However, even if these patients were able to be excluded, we would anticipate larger differences between the definitive therapy and NDT groups. Fourth, more granular detail about medical comorbidities could help clarify specific health-related factors that were associated with the type of therapy received. Fifth, observational studies may be biased because of uncaptured covariates, and the study design limits our ability to infer causation between variables. Sixth, the median follow-up period for this study was 54.9 months, and given the younger age of patients included, longer follow-up is necessary to establish how survival is affected at later time points.

## Conclusions

Despite evidence from large randomized clinical trials demonstrating a survival benefit for definitive local therapy in patients with high-risk prostate cancer, our findings suggest that a significant percentage of younger patients with minimal comorbidities continues to receive NDT. Sociodemographic, health-related, and disease-related factors, including insurance status and race/ethnicity, were associated with receipt of NDT, a decision that was significantly associated with worse survival rates and increased PYLLs. Expanding access to definitive local therapy, including brachytherapy, EBRT, and surgery, for younger patients with high-risk prostate cancer would be anticipated to improve survival.

## Notes

Supplement. **eTable. **Univariate and Multivariate Regression Analyses for Factors Associated With Receipt of Definitive Local Therapy **eFigure 1. **Number of Patients Treated at Individual Treatment Facilities **eFigure 2. **Patterns of Treatment by Facility Type for All Patients and Uninsured Patients **eFigure 3. **Person-Years of Life Lost Based on Insurance Status Among All Patients With High-Risk Prostate Cancer Aged 70 Years and Younger and Those Receiving Definitive Therapy
