# Error in Table 3

**DOI:** 10.1001/jamanetworkopen.2020.5306

**Published:** 2020-04-13

In the Original Investigation titled “Association of Sociodemographic and Health-Related Factors With Receipt of Nondefinitive Therapy Among Younger Men With High-Risk Prostate Cancer,”^1^ published March 19, 2020, there were errors in 2 column headings in Table 3. They should have read, “Nondefinitive therapy: systemic therapy” and “Nondefinitive therapy: no treatment.” This article has been corrected.^1^
