# Supplementary material for: Association of Sociodemographic and Health-Related Factors With Receipt of Nondefinitive Therapy Among Younger Men With High-Risk Prostate Cancer
Source: JAMA Netw Open. 2020 Mar 19;3(3):e201255. doi: 10.1001/jamanetworkopen.2020.1255 (PMC7082722; doi:10.1001/jamanetworkopen.2020.1255)
Supplement: Supplement. — eTable. Univariate and Multivariate Regression Analyses for Factors Associated With Receipt of Definitive Local Therapy eFigure 1. Number of Patients Treated at Individual Treatment Facilities eFigure 2. Patterns of Treatment by Facility Type for All Patients and Uninsured Patients eFigure 3. Person-Years of Life Lost Based on Insurance Status Among All Patients With High-Risk Prostate Cancer Aged 70 Years and Younger and Those Receiving Definitive Therapy [file jamanetwopen-3-e201255-s001.pdf]

Supplementary Online Content

Bagley AF, Anscher MS, Choi S, et al. Association of sociodemographic and health-related factors with receipt of nondefinitive therapy among younger men with high-risk prostate cancer. *JAMA Netw Open.* 2020;3(3):e201255. doi:10.1001/jamanetworkopen.2020.1255

eTable. Univariate and Multivariate Regression Analyses for Factors Associated With Receipt of Definitive Local Therapy

eFigure 1. Number of Patients Treated at Individual Treatment Facilities

eFigure 2. Patterns of Treatment by Facility Type for All Patients and Uninsured Patients

eFigure 3. Person-Years of Life Lost Based on Insurance Status Among All Patients With High-Risk Prostate Cancer Aged 70 Years and Younger and Those Receiving Definitive Therapy

This supplementary material has been provided by the authors to give readers additional information about their work.

© 2020 Bagley AF et al. *JAMA Network Open.*

eTable. Univariate and Multivariate Regression Analyses for Factors Associated With Receipt of Definitive Local Therapy[a]

	**Definitive Therapy**					
	**Univariate**			**Multivariate**		
**Characteristic**	**OR**	**95% CI**	***P***	**OR**	**95% CI**	***P***
**Insurance Status**						
Private insurance/managed care	1 (Reference)			1 (Reference)		
Not Insured	0.23	0.20 – 0.25	<.0001	0.31	0.27 – 0.35	<.0001
Medicaid	0.29	0.26 – 0.33	<.0001	0.43	0.38 – 0.49	<.0001
Medicare	0.72	0.67 – 0.76	<.0001	0.82	0.76 – 0.89	<.0001
Other government	1.96	1.51 – 2.54	<.0001	2.41	1.83 – 3.19	<.0001
**Income Level**[b]						
$63,000+	1 (Reference)			1 (Reference)		
$48,000 - $62,999	0.87	0.80 – 0.94	0.0005	0.87	0.79 – 0.96	0.006
$38,000 - $47,999	0.69	0.64 – 0.75	<.0001	0.75	0.67 – 0.83	<.0001
< $38,000	0.51	0.47 – 0.55	<.0001	0.73	0.65 – 0.82	<.0001
**Race/Ethnicity**						
White (Non-Hispanic)	1 (Reference)			1 (Reference)		
Black	0.44	0.41 – 0.47	<.0001	0.59	0.55 – 0.64	<.0001
Hispanic/Spanish	0.50	0.45 – 0.57	<.0001	0.74	0.65 – 0.84	<.0001
Asian	0.73	0.59 – 0.90	0.003	0.88	0.71 – 1.09	0.23
**Age, yrs**						
≤ 60	1 (Reference)			1 (Reference)		
61 – 65	0.92	0.86 – 0.99	0.02	0.89	0.82 – 0.96	0.002
66 - 70	0.87	0.81 – 0.93	<.0001	0.81	0.74 – 0.89	<.0001

© 2020 Bagley AF et al. *JAMA Network Open.*

**Population without high school degree, %[c]**						
< 7	1 (Reference)			1 (Reference)		
7 - 12.9	0.96	0.88 – 1.04	0.30	1.09	0.99 – 1.20	0.09
13 - 20.9	0.70	0.65 – 0.76	<.0001	1.00	0.89 – 1.11	0.94
> 21	0.51	0.47 – 0.56	<.0001	0.94	0.83 – 1.07	0.33
**Clinical T-Stage**						
1	1 (Reference)			1 (Reference)		
2	1.32	1.24 – 1.42	<.0001	1.29	1.21 – 1.39	<.0001
3	1.92	1.76 – 2.10	<.0001	1.77	1.61 – 1.94	<.0001
4	0.31	0.27 – 0.37	<.0001	0.31	0.26 – 0.37	<.0001
**Charlson Comorbidity Index**						
0	1 (Reference)			1 (Reference)		
1	1.14	1.05 – 1.24	0.002	1.20	1.10 – 1.32	<.0001
2	0.79	0.66 – 0.95	0.01	0.89	0.73 – 1.08	0.23
**Great Circle Distance (miles)**						
0 - 4.9	1 (Reference)			1 (Reference)		
5.0 - 11.4	1.27	1.18 – 1.37	<.0001	1.10	1.01 – 1.19	0.03
11.5 - 27.9	1.50	1.39 – 1.63	<.0001	1.16	1.07 – 1.27	0.0006
> 28.0	1.55	1.43 – 1.68	<.0001	1.29	1.18 – 1.41	<.0001
**Facility Type**						
Academic/Research Program	1 (Reference)			1 (Reference)		
Community Cancer Program	0.78	0.72 – 0.86	<.0001	0.87	0.79 – 0.96	0.005
Comprehensive Community Cancer Program	1.74	1.63 – 1.86	<.0001	1.72	1.61 – 1.85	<.0001
Integrated Network Cancer Program	1.35	1.22 – 1.49	<.0001	1.38	1.24 – 1.53	<.0001
**Urban/Rural Group[d]**						

© 2020 Bagley AF et al. *JAMA Network Open.*

Metro Counties	1 (Reference)			NI		
Urban Counties	1.06	0.98 – 1.15	0.14			
Rural Counties	1.01	0.83 – 1.22	0.93			

[a]A logistic regression model was used for univariate and multivariate analyses. NI, Not included
[b]Median household income for patient's area of residence
[c]Percentage of adults in the patient's zip code who did not graduate from high school
[d]Data estimated by population of patient's county using data from 2013 published by United States Department of Agriculture Economic Research Service. Metropolitan represents counties in metropolitan areas with a population range of fewer than 250 000 to greater than 1 million; urban represents populations of 2500 to greater than 20 000, either adjacent or not adjacent to a metropolitan area; rural represents completely rural populations or areas with less than 2500 residents.

© 2020 Bagley AF et al. *JAMA Network Open.*

eFigure 1. Number of Patients Treated at Individual Treatment Facilities

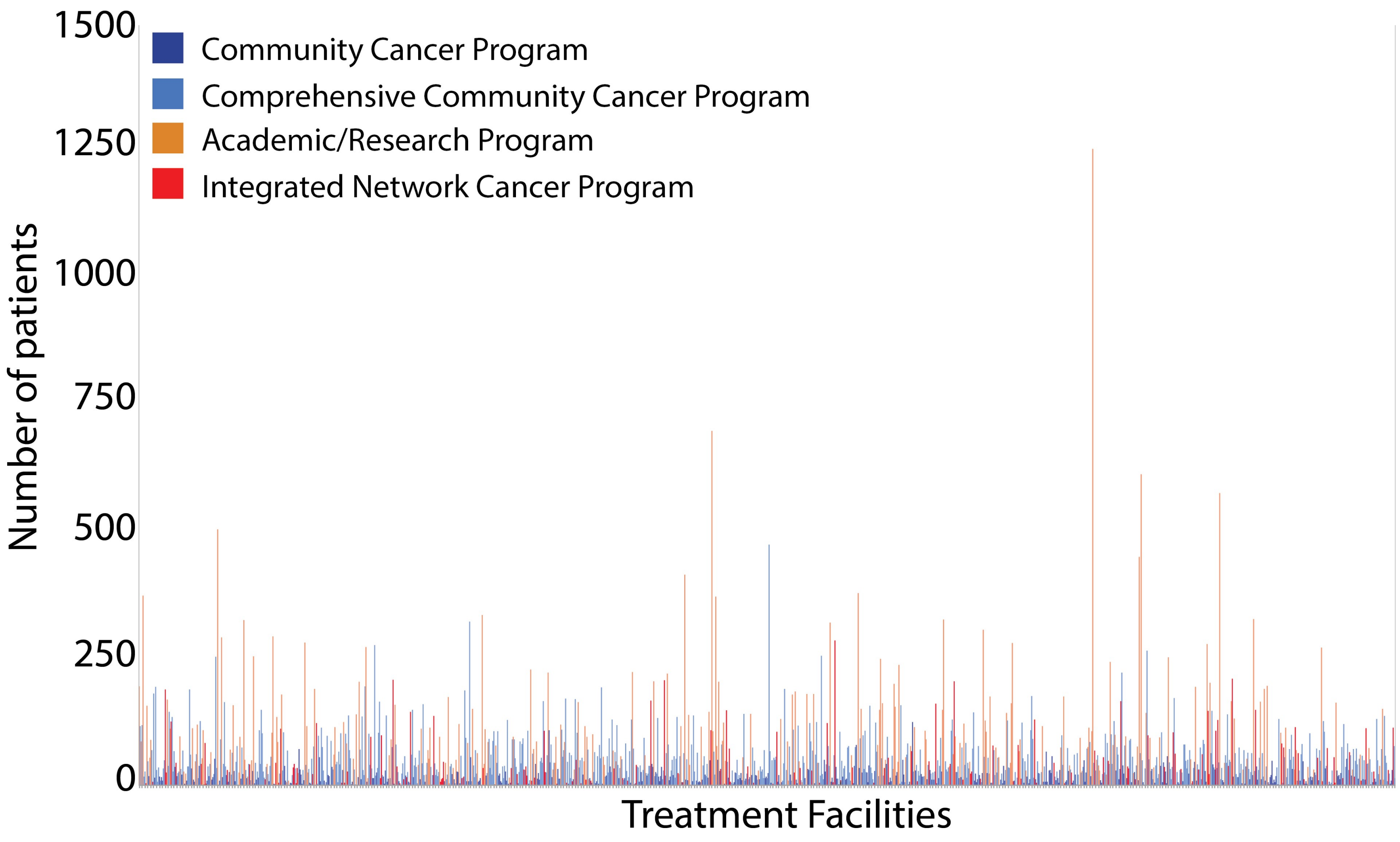

© 2020 Bagley AF et al. *JAMA Network Open.*

eFigure 2. Patterns of Treatment by Facility Type for (A,C) All Patients and (B,D) Uninsured Patients

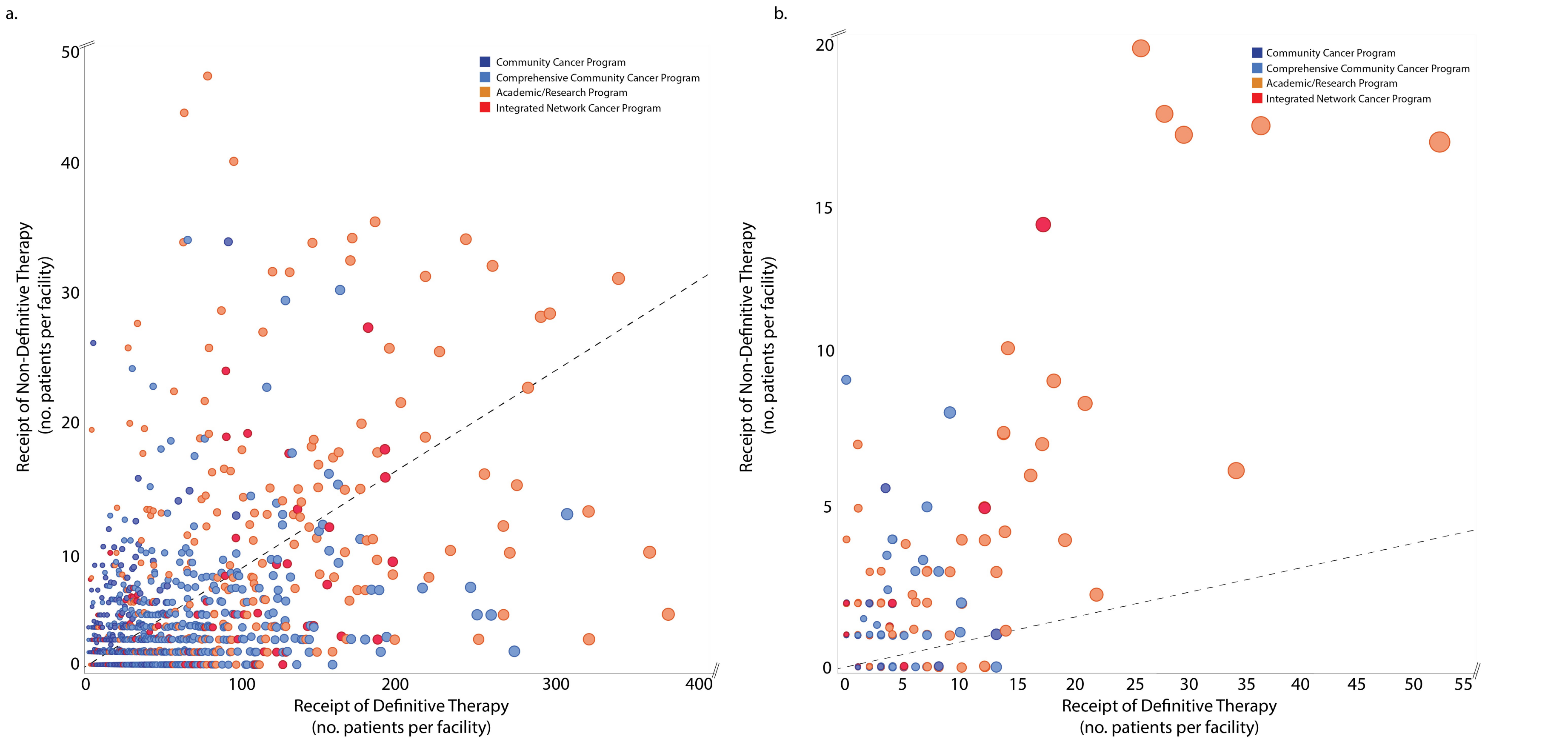

© 2020 Bagley AF et al. *JAMA Network Open.*

eFigure 2. (Continued) Patterns of Treatment by Facility Type for (A,C) All Patients and (B,D) Uninsured Patients

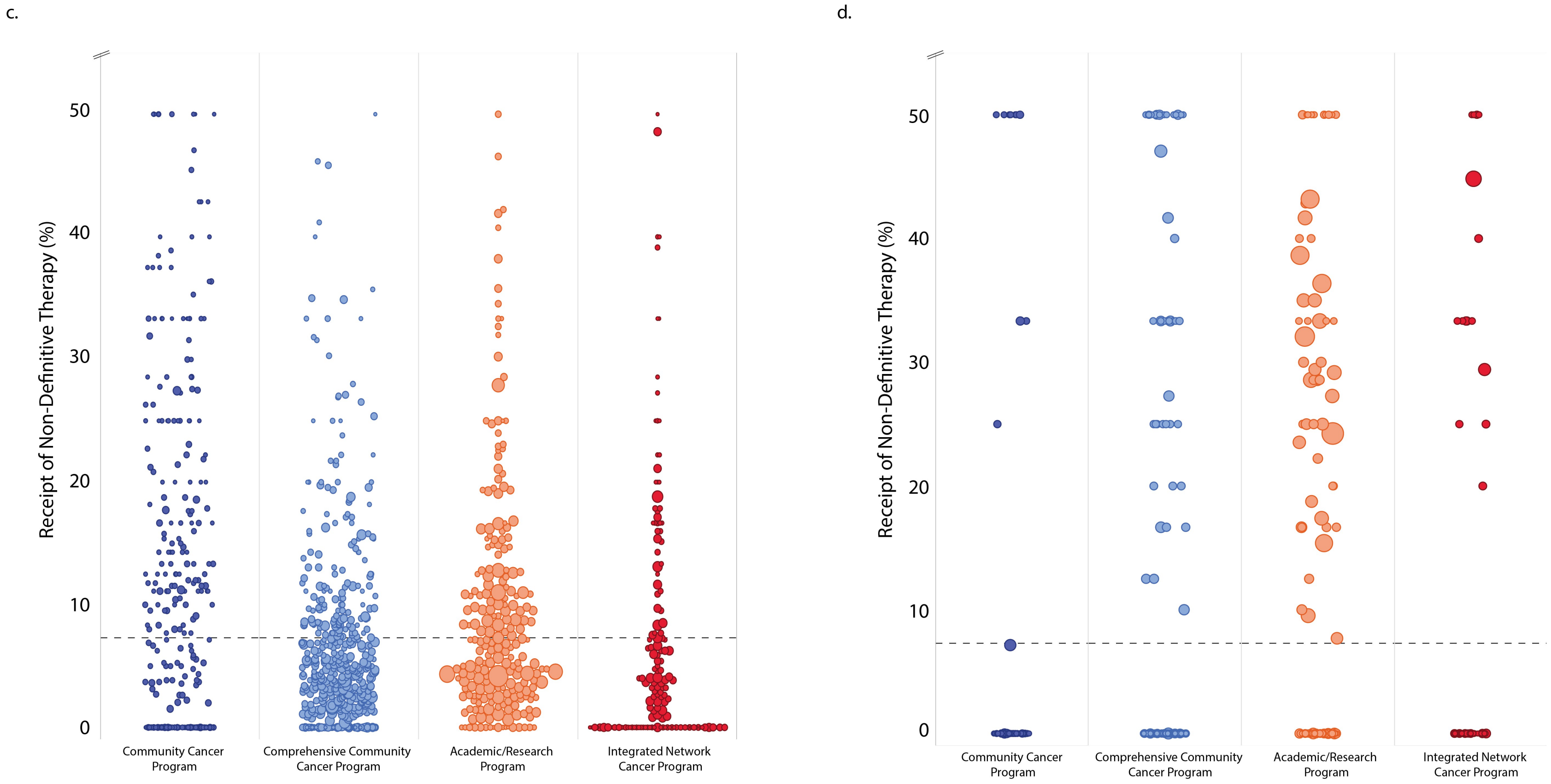

Symbols: Each circle represents an individual treatment facility; color of circle denotes treatment facility type; size of circle is proportional to total number of patients treated at facility; dashed line in (a) – (d) represents rate of non-definitive therapy in overall study population.

© 2020 Bagley AF et al. *JAMA Network Open.*

eFigure 3. Person-Years of Life Lost Based on Insurance Status Among (A) All Patients With High-Risk Prostate Cancer Aged 70 Years and Younger and (B) Those Receiving Definitive Therapy

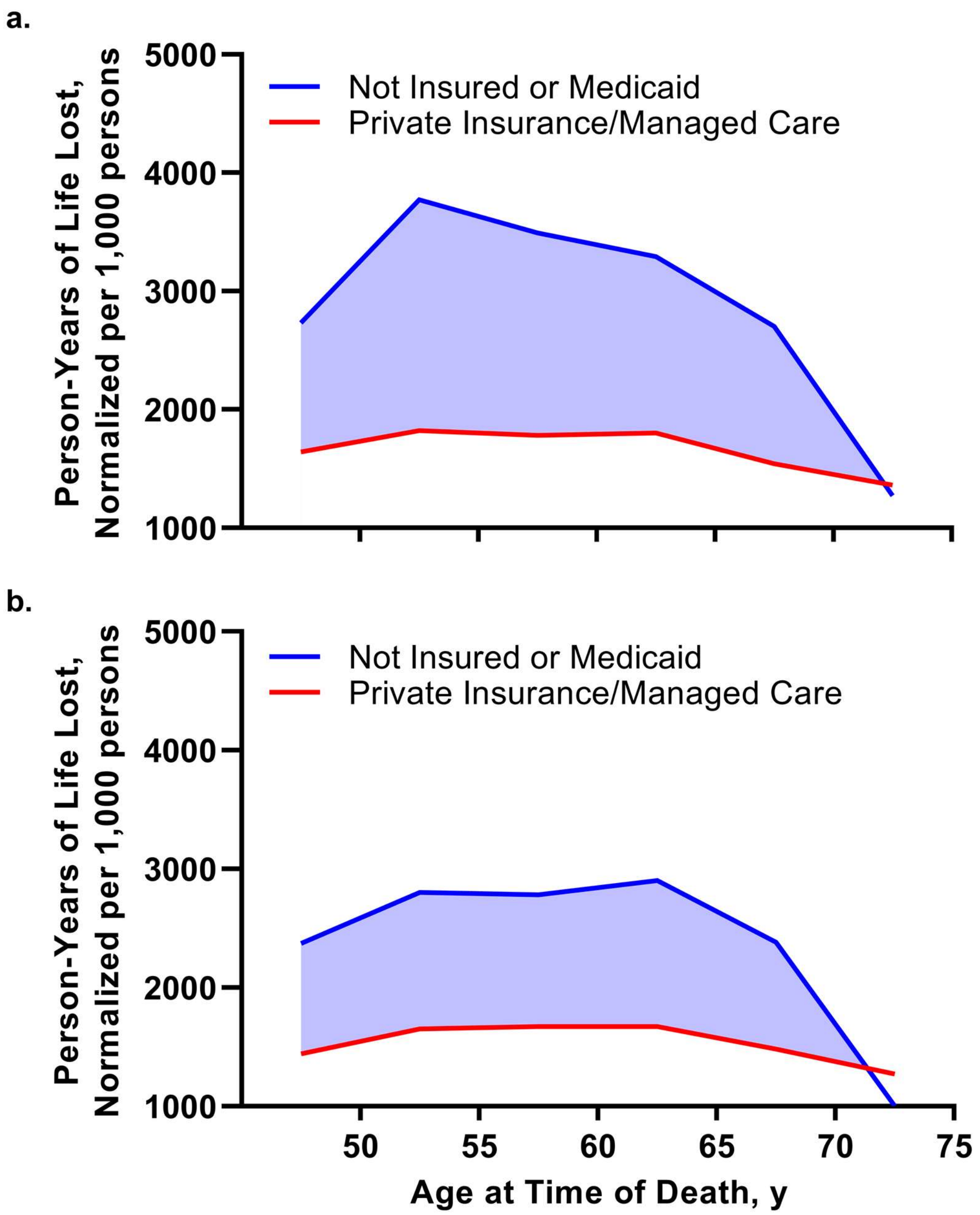

© 2020 Bagley AF et al. *JAMA Network Open.*
